# Surgical Management of Colorectal Liver Metastases: Prognostic Indicators and the Impact of RAS Mutation Status

**DOI:** 10.6004/jadpro.2015.6.5.6

**Published:** 2015-09-01

**Authors:** Steven H. Wei, Leigh A. Samp

**Affiliations:** MD Anderson Cancer Center, Houston, Texas

According to the Centers for Disease Control and Prevention (CDC), colorectal cancer (CRC) is the third most common cancer among men and women and the third leading cause of cancer death among men and women in the United States (CDC, 2014). The liver remains the most common site for metastases, affecting approximately 30% of all patients with CRC and accounting for at least two-thirds of CRC deaths (Abdalla et al., 2006). In patients with liver-only metastases, regional therapies such as hepatic resection, ablation, and chemoembolization may be considered in addition to systemic chemotherapy and as alternatives to systemic therapy alone.

The safety of liver surgery and the feasibility of resection for colorectal liver metastases (CRLM) have been improved by novel operative techniques and innovative multidisciplinary approaches to treatment (Vauthey & Kopetz, 2013). Systemic chemotherapy combined with surgical resection of CRLM improves survival outcomes and is reported to be the most effective approach to the treatment of metastatic disease (Vauthey et al., 2013; Kopetz et al., 2009). Five-year survival after surgery and modern chemotherapy has been reported at upward of 55% for R0 resections (Andreou et al., 2013). Thus, preoperative systemic therapy is increasingly used in this subset of patients to assess the biology of the tumor(s) over time and to aid in the selection of candidates for resection.

One of the main challenges in treating patients with CRLM is to identify those who can derive a significant survival benefit from hepatic resection. Recently, mutations in the Kirsten rat sarcoma viral oncogene homolog (*KRAS*) have received much attention as the most promising mutation for prognostication in patients with CRLM (Karagkounis et al., 2013). Studies indicate that knowledge of *KRAS* mutation status may also prove valuable for surgeons evaluating patients for resection of CRLM.

## Clinical Evaluation

Patients with CRLM present with either synchronous disease (metastasis diagnosed at the same time as primary CRC) or metachronous disease (metastases diagnosed beyond 12 months of initial presentation of CRC). In patients with synchronous disease, a plan to resect the primary tumor must be incorporated into the overall treatment algorithm for liver metastases, thereby increasing the complexity of decision-making in these situations.

At many institutions, patients with CRLM initially consult with the hepatobiliary team, which generally includes evaluation by a liver surgeon, a colorectal medical oncologist, and advanced-practice providers. In addition to a full laboratory panel (Table 1), patients with CRLM should undergo cross-sectional imaging sufficient to ascertain the distribution of disease within the liver.

**Table 1:**
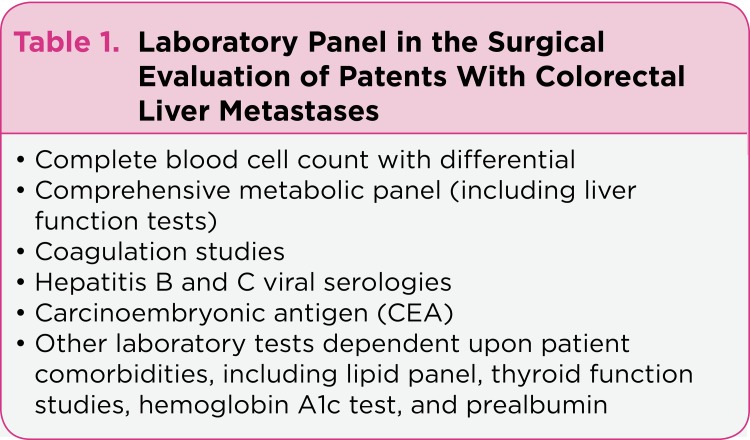
Laboratory Panel in the Surgical Evaluation of Patents With Colorectal Liver Metastases

Preferred imaging modalities include either a multiphasic "liver protocol" computed tomography (CT) or magnetic resonance imaging (MRI). Positron-emission tomography (PET) scans are occasionally obtained to rule out extrahepatic disease but should not be utilized as the sole imaging modality upon which to base surgical resectability of CRLM.

Because complete resection of the liver lesions should account for all known sites of disease, a thorough review of the patient’s pretreatment imaging is critical to determine the true extent of disease prior to the administration of systemic chemotherapy (Adams et al., 2013). A detailed map of the liver lesions, including the involved anatomic segments, vessels, and bile ducts, helps guide the surgical and medical oncology teams to consider treatment with either a neoadjuvant systemic chemotherapy regimen or upfront hepatectomy in select patients with resectable disease.

## Presurgical Strategies in CRLM

When considering long-term survival for patients with CRLM, hepatic resection is the preferred method of treatment, provided that all known tumors can be surgically removed with clear margins and the remnant liver volume is sufficient to perform all necessary functions. (See Table 2 for definition of complete resection of tumor-bearing liver.)

**Table 2:**
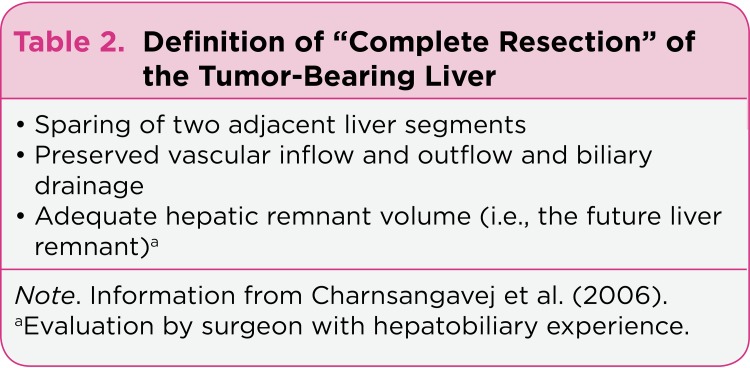
Definition of "Complete Resection" of the Tumor-Bearing Liver

There are several strategies used by liver surgeons to help prepare patients for potentially curative hepatic resection. Patients with a small future liver remnant (FLR) volume (less than 20% of the total estimated liver volume) may benefit from preoperative portal vein embolization (PVE) to induce hypertrophy of the FLR in an effort to decrease the risk of hepatic insufficiency or failure following major hepatic resection (Vauthey et al., 2013; Adams et al, 2013). In patients with multiple, bilateral liver metastases, complete resection may be feasible with a multidisciplinary surgical strategy involving the use of perioperative systemic chemotherapy, PVE, and staged hepatectomies (Brouquet et al., 2011).

Furthermore, other regional therapies such as percutaneous liver ablation, chemoembolization, and/or radioembolization may play a complementary role in helping to eradicate liver tumors; they can be used either alone or as a bridge to surgery. With the utilization of more effective systemic chemotherapy, a larger percentage of patients with CRLM are responding and becoming potential candidates for curative liver resection.

## The Era of Modern Chemotherapy

Since the use of single-agent fluorouracil (5-FU) through the 1990s, there has been a dramatic increase in the number of available chemotherapeutic regimens for CRLM. Today, the majority of patients receive sequential, multimodality therapy with oxaliplatin- or irinotecan-based chemotherapy, along with the addition of certain targeted agents such as the antivascular endothelial growth factor (VEGF) agent bevacizumab (Avastin) or the epidermal growth factor receptor (EGFR) inhibitor cetuximab (Erbitux; Vauthey et al., 2013).

**Figure 1:**
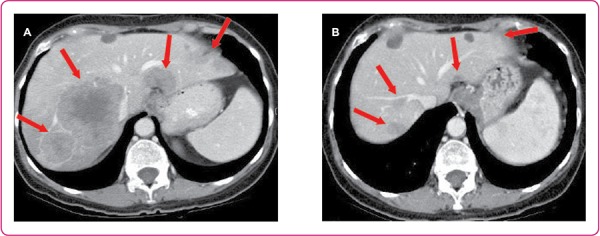
Images of bilateral colorectal liver metastases (A) before and (B) after four courses of neoadjuvant systemic chemotherapy (e.g., modified FOLFOX [folinic acid, fluorouracil, and oxaliplatin] and bevacizumab). Images courtesy of Dr. Jean-Nicolas Vauthey.

**Figure 2:**
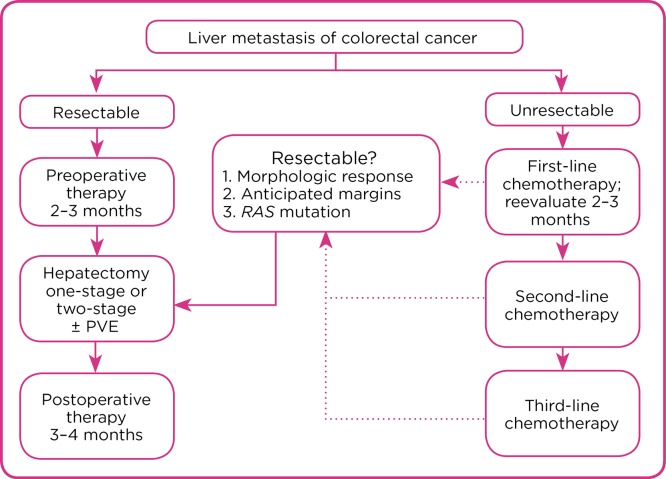
Multidisciplinary treatment algorithm for patients with liver metastases from colorectal cancer. PVE = portal vein embolization. Adapted from Kopetz & Vauthey (2008).

The use of modern chemotherapy with targeted biologic agents has been shown to improve overall response rates, allowing more patients with CRLM to become potential candidates for curative liver resection (Figure 1; Kopetz et al., 2009). Multidisciplinary collaboration between the liver surgeon and the medical oncologist (see Figure 2) remains crucial in determining the proper chemotherapy regimen and duration of treatment for patients with potentially resectable CRLM prior to hepatic resection.

## Prognostic Indicators for CRLM

One of the main challenges in treating patients with CRLM is to identify those who can derive a survival benefit from hepatic resection. Historically, predictors of survival were based on several clinical parameters, such as age, primary tumor location, nodal status, number and size of liver metastases, carcinoembryonic antigen (CEA) level, and disease-free interval (DFI) between detection of the primary tumor and development of metastases (Nordlinger et al, 1996). In the 1990s, various scoring systems were published to predict prognosis after resection of CRLM (Fong, Fortner, Sun, Brennan, & Blumgart, 1999; Rees, Tekkis, Welsh, O’Rourke, & John, 2008).

However, in recent years, several studies have questioned the validity and clinical application of these risk scores, since the prognostic value of many of these factors was determined before the emergence of effective preoperative chemotherapy (Zakaria et al., 2007; Kattan et al, 2008). With the increased use of preoperative chemotherapy, both pathologic and radiographic responses have been found to be strong independent predictors of survival and are associated with long-term outcomes in patients with CRLM (Blazer et al., 2008; Chun et al., 2009).

Recent advances in chemotherapy, along with improved surgical techniques have increased the proportion of patients benefiting from curative resection of CRLM. However, there continues to be a diverse set of outcomes, including the rapid onset of liver recurrence and development of extrahepatic disease, in some patients. In patients with CRLM, there remains much variability in clinical presentation, biologic aggressiveness, and patterns of treatment failure, and no known biomarker can predict these differences. Evaluation of molecular tumor characteristics, including somatic gene mutations, may provide additional insight into tumor biology and useful information to guide treatment for patients with CRLM.

## The Emerging Role of *RAS* Mutation Status

Due to the complexity of the genomic landscape of CRC, researchers have focused on cell-signaling pathways, rather than individual genes, to provide insight into the course of tumorigenesis. The signal transduction downstream from EGFR affects two intracellular pathways relevant in CRC, and both are controlled by KRAS, their common upstream regulator. Gene mutations affecting the binding activities of the EGFR ligand may cause dysregulation of the pathways, which can lead to cell proliferation, inhibition of apoptosis, angiogenesis, cell migration, adhesion, and invasion (Berg & Soreide, 2012). Recent studies have found that mutated *KRAS* is present in 35% to 45% of patients with CRLM, hence its relevance in patients with advanced-stage CRC (Nash et al., 2010; De Roock et al., 2010).

Over the past decade, mutation status of *RAS* family genes (predominantly *KRAS* and *NRAS*) has been shown to correlate with the effectiveness of anti-EGFR agents for unresectable metastatic CRLM (Van Cutsem et al., 2009). Currently, medical oncologists test for molecular biomarkers and use the mutation status of the *KRAS* gene to select patients with advanced-stage CRC with wild-type *KRAS* for treatment with a monoclonal antibody targeting EGFR (Amado et al., 2008). In a study by Nash et al, somatic gene mutations of the *KRAS* oncogene were found to be associated with negative outcomes after resection of CRLM, predating the era of modern chemotherapy (Nash et al., 2010).

In a study by Karagkounis et al. (2013), *KRAS* mutation status was found to be an independent predictor of overall survival (OS) and recurrence-free survival (RFS) following surgery for CRLM. The *KRAS* gene mutation was detected in 29% of patients with CRLM, which is lower than that reported in most other studies. The lower rate of *KRAS* mutation in the Karagkounis study (2013) may be attributed to the selection of patients, who were deemed candidates for liver surgery and, therefore, were more likely to have a better tumor biology and longer survival than those with unresectable CRLM.

It has also been reported that patients with extrahepatic metastases from CRC have higher rates of *KRAS* mutation than patients with CRLM alone (Tie et al., 2011). Since the presence of extrahepatic metastases is generally considered a contraindication for resection of CRLM, it is reasonable to expect a lower rate of *KRAS* mutations among patients undergoing liver resection for CRLM.

Although *KRAS* is the most common somatic gene mutation in patients with CRLM, both *V600E* mutation of the *BRAF* gene status and *NRAS* mutation status have also been considered for analysis. Several studies have demonstrated that the presence of the *V600E* mutation is associated with a poor prognosis and worse OS in patients with CRLM (De Roock et al., 2010; Tran et al., 2011).

In the study by Karagkounis et al. (2013), the presence of the *V600E* mutation of the *BRAF* gene was found in only 4 of 202 patients (2%); therefore, no definitive association between *BRA*F mutation status and survival outcomes could be made. In a study by Vauthey et al. (2013), patients with *RAS* mutations had worse long-term outcomes than those with wild-type *RAS*. The addition of *NRAS* mutation increased the yield of all *RAS* mutations by 20%, thereby strengthening the impact of mutations on prognosis.

## *RAS* Mutation Status as a Prognostic Marker for CRLM

Among all the molecular biomarkers tested in patients with CRLM, the *KRAS* oncogene stands out for two important reasons. First, *KRAS* mutation is consistently associated with primary and secondary resistance to EGFR-directed therapy, and second, it demonstrates use as a strong prognostic indicator for patients undergoing surgical therapy for CRLM.

**Figure 3:**
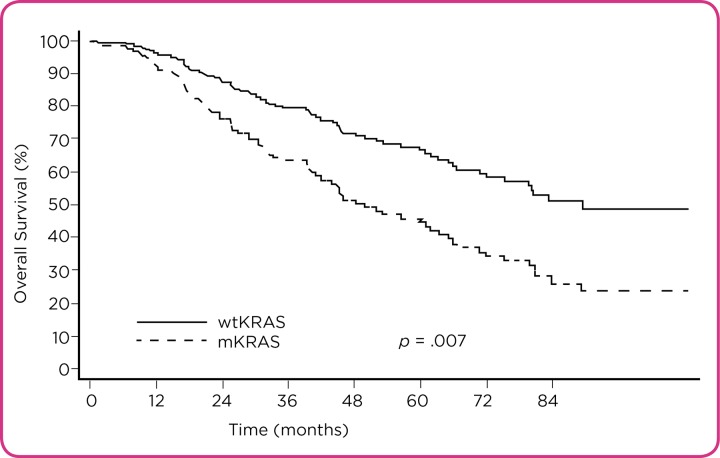
Overall survival after hepatic surgery for colorectal liver metastasis depicted by KRAS mutation status following multivariate Cox model. wtKRAS = wild-type KRAS; mKRAS = mutant KRAS. Reprinted from Karagkounis et al. (2013) with permission from Wiley.

In the Karagkounis study (2013), the median survival among patients with mutant *KRAS* was 45.2 months, compared with 71.9 months for patients with wild-type *KRAS*, with 5-year OS of 49.8% and 57.4%, respectively. After adjusting for known predictors of survival, in multivariate analysis, mutant *KRAS* was independently associated with worse OS after resection of CRLM (Figure 3). Furthermore, patients with mutant *KRAS* demonstrated a worse RFS (median 11.8 months vs. 20.8 months for patients with wild-type *KRAS*).

In another study, Vauthey et al. (2013) investigated the pattern of recurrence after resection of CRLM. They found that *RAS* mutation was a strong predictor of lung but not liver recurrence. Several other studies have confirmed the correlation among the presence of *KRAS* mutations, poorer patient survival outcomes, and recurrence patterns following curative liver surgery (Vauthey et al., 2013; Nash et al., 2010; Tran et al., 2011; Kawamoto et al., 2012).

## Conclusion

Although liver surgery remains the only curative option for patients with CRLM, the majority of patients will experience recurrence within 5 years of resection. With recent advances in chemotherapy and improvements in surgical techniques, more patients are benefiting from hepatic resection.

Various clinicopathologic factors have been associated with prognosis; however, there are few biomarkers that predict which patients may have more aggressive disease or even which patients may derive the greatest benefit from surgical therapy. Studies have shown that *KRAS* mutation status has prognostic value as an "early-event" biomarker for patients undergoing resection of CRLM, regardless of chemotherapy regimen, and should be considered in the evaluation of patients undergoing liver resection for CRLM (Kawamoto et al., 2012).

In practice, the presence of the *KRAS* mutation alone should not exclude patients from surgery, but a finding of wild-type *KRAS* may encourage pursuance of more aggressive treatment in patients with borderline resectable disease. Along with other clinicopathologic predictors, *KRAS* mutation status is clearly useful in early treatment planning—both in the preoperative assessment of patients with CRLM and during follow-up—to assess the risk of recurrence and long-term survival. The analysis of *RAS* mutation status in patients with CRLM may represent a first step in a personalized evaluation of this disease with molecular data.

Advanced practitioners in both surgical and medical oncology play key roles in the management and treatment of patients with CRLM. They are often the first clinical providers seen by these patients soon after their diagnosis, and many are also responsible for ordering and interpreting the appropriate tests, including molecular gene panels and presurgical imaging studies.

It is important for advanced practitioners to understand the potential implications of biomarkers in CRLM and other prognostic indicators. Patients often rely on advanced practitioners to help explain complex disease processes, including staging, treatment plan, and prognosis, so they can make informed decisions about their own care. Advanced practitioners need to educate themselves and communicate with colleagues who share the care of these patients to lead to improved patient counseling and more efficient coordination of care.
